# Serological Evidence of Exposure to *Leptospira* spp. in Veterinary Students and Other University Students in Trinidad and Tobago

**DOI:** 10.1155/2013/719049

**Published:** 2013-01-09

**Authors:** Ambrose James, Kingsley Siele, Neeka Harry, Sharianne Suepaul, Alva Stewart-Johnson, Abiodun Adesiyun

**Affiliations:** School of Veterinary Medicine, Faculty of Medical Sciences, University of the West Indies, Saint Augustine, Trinidad and Tobago

## Abstract

The study compared the serological evidence of leptospirosis in 212 students in four schools (veterinary, dental, advanced nursing education and pharmacy) of the University of the West Indies (UWI), by testing for IgG immunoglobulins to *Leptospira* spp. using the enzyme-linked immunosorbent assay (ELISA) and the microscopic agglutination test (MAT). Overall, of 212 students tested by the ELISA, 12 (5.7%) and 31 (14.6%) were positive and borderline, respectively. Amongst the 113 veterinary students 11 (9.7%) and 19 (16.8%) were seropositive and borderline respectively compared with nonveterinary students with corresponding values of 1 (1.0%) and 12 (12.1%). The frequency of serological evidence of leptospirosis by the ELISA was statistically significantly (*P* < 0.05; *χ* ^2^) higher in veterinary students, 26.5% (30 of 113) than in nonveterinary students, 13.1% (13 of 99). By the MAT, the seropositivity for leptospirosis was similar for veterinary students, 7.1% (8 of 113) and nonveterinary students, 7.1% (7 of 99). For veterinary students, the prevalent infecting serovar was Icterohaemorrhagiae Copenhageni while amongst nonveterinary students, the prevalent serovar was Australis Rachmati. Being a veterinary student was the only risk factor that was significantly associated with *Leptospira* infection indicating that veterinary students need to be cognizant and to practise preventive measures for leptospirosis.

## 1. Introduction

Leptospirosis is a bacterial zoonosis with global distribution, although it has been documented in developing and developed as well as temperate and tropical countries [1, 2]. This disease occurs predominantly as a subclinical infection although cases of clinical disease with numerous nonpathognomonic signs and symptoms have been reported [1–4]. It is therefore responsible for morbidities and mortalities worldwide [2, 5, 6].

 It has been established that rodents are primary reservoirs for human and animal infections by *Leptospira *spp. which is one of the reasons that the prevalence of leptospirosis is higher in tropical environments with high rainfall and humidity and prevalent poor sanitary conditions which support the proliferation of rodents [7–9]. The distribution of the primary reservoir and the ability of the pathogen to infect animals (livestock, wildlife, pet animals, and others) have made leptospirosis an occupational disease [9–11]. 

 High risk individuals include livestock farmers, animal handlers, veterinarians, slaughter house workers, sewerage or environmental sanitation workers, sugar cane, and rice field workers, compared to members of the general population [9, 11, 12]. Human infections are known to result from direct and indirect contact with urine of rodents or other animals containing high numbers of viable leptospires or following consumption of contaminated food or water [13, 14]. A number of factors, in addition to occupational exposure, have been reported to affect exposure potential in humans to leptospirosis. These factors include the age, gender, season of the year, and geographical locations and have been known to affect the infection rate in humans [15, 16]. 

 Diagnosis of human leptospirosis can be achieved through the demonstration of the microorganism itself or by the detection of antibodies produced against the pathogen following infection [13, 17]. The organism can be demonstrated by culture in growth media, special staining of infected tissues, and the use of dark-field microscopy [13, 18]. 

 There is a wide variety of serological tests which can detect IgG, IgM, and IgA [19]. Some of these tests include the enzyme-linked immunosorbent assay (ELISA) [20] and microscopic agglutination test (MAT) [21] amongst others. The MAT is considered the “gold standard” for the serological diagnosis of leptospirosis and also for the serotyping of *Leptospira* isolates [21, 22]. The advantages and disadvantages of the MAT as a diagnostic test are well documented in the literature [21–23]. The ELISA is easy to perform, rapid, with a high sensitivity, and amenable to be standardized but the specificity is low and it is genus-specific and, therefore, unlike the MAT, cannot be used to serotype infecting leptospires [24, 25]. 

 In Trinidad and Tobago, several reports exist on the prevalence of leptospirosis with the detection or isolation in school children [26], apparently healthy individuals [27] and in piggery farm workers [28]. Mohan et al. [29] conducted a retrospective study which determined the average annual incidence rate of leptospirosis and indicated that rate was affected by season, gender and age. In a study conducted on sugarcane field workers, Adesiyun et al. [30] reported the seroprevalence of leptospirosis to be 0.7%. To date there is no report on the occurrence of leptospirosis in students at tertiary institutions in the country. 

 The specific objectives of the current study were to compare the frequency of serological evidence of leptospirosis in veterinary students with nonveterinary students in the Faculty of Medical Sciences, to determine the infecting serovars of *Leptospira *spp. and to investigate the important risk factors for leptospirosis in these students.

## 2. Materials and Methods

### 2.1. Student Population Studied

The study group comprised students in four schools, School of Dentistry (SOD), School of Veterinary Medicine (SVM), School of Advanced Nursing Education (SANE), and School of Pharmacy (SOP), at the Faculty of Medical Sciences (FMS). The study was conducted from August 2010 to July 2011 when the student population in each of the schools were as follows: SOD (160), SVM (170), SOP (279), and SANE (89), a 3-year programme.

### 2.2. Study Design

The study design involved the sampling of students of each of the schools who volunteered to participate and then comparing the serological evidence of veterinary students with those of nonveterinary students. 

### 2.3. Determination of Sample Size

The sample size for the student population will be determined using the formula *n*_*o*_ = *t* ^2^ × *p*(1 − *p*)/*d* ^2^ [31], where *t* = 1.96, *d* = precision at type 1 error of 0.04, *p* = prevalence = 11% [28], and *n*_*o*_ = estimated sample size. Since this equation is based on an infinite population, *n*_*o*_ was adjusted to suit a definite population of 698 student population in four schools (SOD, SVM, SANE, and SOP) in the FMS, using the formula *n*_adj_ = (*N* × *n*_*o*_)/(*N* + *n*_*o*_), where *n*_adj_ was the number of humans required to estimate prevalence at the same absolute precision as the first equation. The estimated sample size used in the current study was therefore: *n*_*o*_ = 1.96^2^ × 0.11(1 − 0.11)/0.04^2^ = 3.84 × 0.0979/0.0016 = 235 and the minimum adjusted sample size was *n*_adj_ = (235 × 698)/(235 + 698) = 176.

### 2.4. Administration of Questionnaire

A questionnaire was administered to each participant in order to obtain information including the programme of study, year in the programme, age, gender, place of residence, ownership of pet animals (dogs, cats, and rodents), association with livestock, farming activities outside the FMS, and other risk factors. Each participant was allowed to pick a random number which was used to identify the samples and the test results of the participant. To maintain the confidentiality of the study, e-mail addresses were obtained to convey the results of the study to each participant.

### 2.5. Collection of Samples

Qualified phlebotomists and nurses from the SANE assisted in blood collection from participants. Approximately 5 mL of blood was drawn from either the median cubital or cephalic veins of the arm using a 21-gauge one and a half inch needle attached to a 5 mL syringe. The blood was then placed into tubes without anticoagulant. Collected blood was refrigerated overnight at 4°C after which it was centrifuged and serum harvested and stored at −20°C until tested. This was a serological study to detect exposure experience of *Leptospira *spp. amongst the students studied using both the ELISA and MAT and therefore no attempt was made to culture the blood samples for *Leptospira* spp.

### 2.6. Assay for Immunoglobulins to *Leptospira* spp. Using ELISA and MAT

The captureELISA to detect IgG (existing infection) was used to detect prior exposure of participants to leptospirosis in microtitre plates as stipulated by the manufacturer (SERION ELISA *classic Leptospira *IgG/IgM, Hersteller Manufacturer Fabricant, Friedrich-Berguis-Ring 19D, 97076 Wurzburg, Germany). The assay with appropriate controls was performed as stipulated by the kit manufacturer. The concentration of IgG (units per mL) in samples was determined using the standards provided in the test kit. These samples were then classified as follows: negative: 0 to 4.9 units/mL., borderline: 5.0 to 9.9 units/mL, and positive: 10 units and higher as recommended for the test kit. 

For the microscopic agglutination test (MAT), 26 serovars of lyophilized antigens were obtained from the Koninklijk *Instituut voor de Tropen*/Royal Tropical Institute (KIT), Biomedical Research Laboratory, Amsterdam, The Netherlands. The international panel utilized consisted of the following serogroups/serovars: Australis Bratislava Jez Bratislava, Ballum Ballum Mus 127, Canicola Canicola Hond Utrecht IV, Grippotyphosa Grippotyphosa Duyster, Grippotyphosa Grippotyphosa Mandemakers, Hebdomadis Hebdomadis Hebdomadis, Icterohaemorrhagiae Icterohaemorrhagiae Kantorowic, Icterohaemorrhagiae Copenhageni Wijnberg, Pomona Pomona Pomona, Pomona proechimys 1161 U, Sejroe Hardjo Hardjoprajitno, Sejroe Saxkoebing Mus 24, Sejroe Sejroe M 84, Semaranga Patoc Patoc I, Andaman Andaman Ch 11, Australis Australis Ballico, Autumnalis Rachmati Rachmat, Bataviae Bataviae Swart, Cynopteri Cynopteri 3522 C, Panama Panama CZ 214 K, Pyrogenes Pyrogenes Salinem, Semaranga Semaranga Veldrat Sem 173, Shermani Shermani 1342 K, Autumnalis Bim, Icterohaemorrhagiae Mankarso, and Tarassovi Tarassovi Perepelicin. The MAT used consists of two parts, qualitative to detect the serogroups present in the antisera and the quantitative aspect to determine the titres of antibodies present. For the qualitative aspect, which followed the protocol described by World Health Organization (13), the panel of 26 serovars were used. The antigens were subcultured in Bijou bottles weekly, incubated at 30°C and checked for the attainment of a density of 1-2 × 10^8^ after 5–7 days. For determination of titres (the quantitative part), serial 2-fold serum dilutions were made with any sera showing agglutination to any of the antigens used. Where there were 2 serovars with the same titre, these were considered as mixed infections. This procedure was done following standard protocol as described by the Royal Tropical Institute in the manual for the international course on laboratory methods for the diagnosis of leptospirosis (21). A titre of 1 : 20 and higher was considered as evidence of *Leptospira *spp. infection as recommended for apparently healthy humans [32].

### 2.7. Approval of Study by Ethics Committee

The Ethics Committee of the Faculty of Medical Sciences approved the study prior to commencement. The objectives and protocol for the study were explained to all the participants and a written consent was obtained. 

### 2.8. Data Analyses

The Statistical Package for Social Sciences (SPSS) version 15.0 was used to produce 2 × 2 contingency tables. Chi-square analysis was conducted to determine risk factors for leptospiral infection in humans and level of significance will be fixed at alpha = 0.05.

## 3. Results

### 3.1. Demographic Data of Students Studied

Table 1 shows the demographic data of students from the four schools in the FMS, University of the West Indies, Trinidad and Tobago, who volunteered to participate in the study. Overall, a total of 212 students participated in the study comprising 113 veterinary students and 99 nonveterinary (46 dental, 39 nursing, and 14 pharmacy) students. Based on data generated on students' characteristics and risk factors for leptospirosis, a majority (54.7%) of students sampled were in years 2 and 3 of their respective programmes, aged 25 years and older (37.3%), and female (73.1%) and reside at homes (53.3%). Amongst the participants across the four schools, none (0.0%) had prior diagnosis of leptospirosis, 69.8% kept pet animals (dogs/cats) with the highest frequency amongst veterinary students (80.5%). A majority (41.0%) kept animals strictly as home or pet animals; the most (36.8%) maintained low levels of contact with their animals with the exception of veterinary students where a majority (46.9%) mentioned that they have close contact with their pets. A high frequency (98.6%) of pet animals did not have recent diagnosis of leptospirosis. Contact with livestock was highest (31.0%) amongst veterinary students. Also the highest frequency of farming (10.6%) outside the faculty was practised by veterinary students of which most were livestock farming (9.7%). A total of 83 (39.2%) of 212 students stated that they encountered rodent problem in the premises where they lived but only 3.3% keep rodents as pets.

### 3.2. Frequency of Detection of Immunoglobulin to *Leptospira* by the ELISA

Of the 113 veterinary students tested, using the ELISA, IgG immunoglobulins to *Leptospira *spp. 11 (9.7%) and 19 (16.8%) were classified as positive and borderline results, respectively, while for nonveterinary students it was 1 (1.0%) and 12 (12.1%), respectively, as shown in Table 2. The differences were statistically significant (*P* < 0.05; *χ* ^2^). Overall, a comparison of the seropositivity rate for IgG immunoglobulins (positive and borderline) to *Leptospira* spp. in veterinary students, 26.5% (30 of 113), was statistically significantly (*P* < 0.05; *χ* ^2^) higher than for nonveterinary students from the other schools, 13.1% (13 of 99).

### 3.3. Frequency of Detection of Immunoglobulin to *Leptospira* by the MAT

The seropositivity rates for immunoglobulins to* Leptospira* spp. when assayed by the MAT were similar for veterinary students, 7.1% (8 of 113), and nonveterinary students, 7.1% (7 of 99), as shown in Table 3. Only seven (26.9%) of the 26 serovars tested were agglutinated at a titre of 20 and higher. For the 16 significant agglutinations detected, 7 (43.8%), 7 (43.8%), 1 (6.3%), and 1 (6.3%) were at titres 1 : 20, 1 : 40, 1 : 80, and 1 : 320, respectively. Five (83.3%) of the 6 significant agglutinations of serovar Icterohaemorrhagiae were detected amongst veterinary students while all 5 (100.0%) significant agglutinations of serovar Australis Rachmati were amongst nonveterinary students.

### 3.4. Comparison of the ELISA and MAT Results

A comparison of both serological tests used in the study showed that the frequency of serological evidence in all students was significantly (*P* < 0.05; *χ* ^2^) higher, 20.3% (43 of 212), by the ELISA compared with the 7.1% (15 of 212) detected by the MAT.

### 3.5. Risk Factors for Seropositivity for Immunoglobulins to *Leptospira*

Regarding seropositivity by risk factors, enrolment as a veterinary student was the only factor that was statistically significantly (*P* < 0.05; *χ* ^2^) associated with serological evidence of leptospirosis and this was detected only using the ELISA. Notably, the other risk factors studied (year in the programme, age, gender, place of residence, recent diagnosis of leptospirosis in pet animals or their owners, ownership of pets (dogs, cats, and rodents), rodent problem at/around homes, class of dogs owned (strictly pet, strictly guard, or both), and contact with pet animals or livestock (low, medium, or high) farming (livestock, rice, or sugarcane) were not significantly (*P* > 0.05; *χ* ^2^) associated with seropositivity for *Leptospira *spp. infection.

## 4. Discussion

 A seroprevalence of 9.7% detected amongst apparently healthy veterinary students using the IgG ELISA in the current study is comparable to the 8.14% reported for veterinary students in Spain [33] where the IgG ELISA was similarly used. However, in that study it was established that the rate of infection by *Leptospira* spp. increased with the number of years enrolled in the veterinary programme contrary to what was found in our study where the seropositivity rate was not significantly different for students by class in the programme. This may be due, in part, to the fact that from the first year and throughout the programme veterinary students are exposed to skills training and an externship programme which bring them in close contact to animals. Animal handling and exposure have been reported to be important in contracting *Leptospira* spp. infection [9, 11, 12, 33, 34].

 The finding, by the use of the ELISA that the serological evidence of exposure to *Leptospira *spp. in veterinary students was statistically significantly higher than found in nonveterinary students therefore did not come as a surprise. In the current study, being a veterinary student was in fact the only risk factor that was determined to be significantly associated with exposure to *Leptospira *spp. 

The relatively low titres (mostly 1 : 20 and 1 : 40) detected by MAT known to have high specificity and low sensitivity [21–23] may have been responsible for the low seropositivity rate detected by the MAT. In apparently healthy individuals, low titres ranging from 1 : 20 to 1 : 40 have been used to determine seropositivity for leptospirosis [32, 33]. 

 Of diagnostic significance is the finding that the ELISA detected significantly higher frequency (20.3%) of serological evidence of exposure experience of *Leptospira *spp. in all the students studied compared with the MAT (7.1%). This finding agrees with published reports where the ELISA has been documented to have higher sensitivity and lower specificity than the MAT [20, 24, 25]. Another reason that may be responsible, in part, for the lower sensitivity of the MAT is the number and type of serovars of *Leptospira *spp. in the panel used for testing [22, 35]. It has been reported that the use of serovars prevalent in a particular geographical area in the screening panel of serovars increases the sensitivity of MAT [21–23]. A major advantage of the MAT over the ELISA is however the fact that it is able to determine the serovars of the infecting *Leptospira* spp. while the ELISA is genus specific. It is also pertinent to mention that cross-reactions may occur across serovars but it is also known that individuals may be exposed to multiple serovars of *Leptospira *spp. and therefore may not be necessarily due to cross-reaction amongst serovars.

 Of epidemiological significance was the finding that the predominant *Leptospira* serovar to which veterinary students have been exposed was Icterohaemorrhagiae Copenhageni while, for nonveterinary students, serovar Australis Rachmati was the most frequently detected. This is relevant because most recent studies using isolation and serological techniques demonstrated that serovar Icterohaemorrhagiae Copenhageni was most prevalent in dogs (cases of clinical leptospirosis, apparently healthy stray and pet dogs), wild rats, and livestock [35–37]. It is therefore obvious that this serovar which is important in causing clinical leptospirosis and subclinical infections in apparently healthy animals is also most likely responsible for the seropositivity for *Leptospira* spp. in veterinary students in the country, emphasizing the zoonotic significance of leptospirosis. It was of interest that serovar Australis Rachmati, not detected in veterinary students, was most common in nonveterinary students from other schools. The implication of this finding is not readily apparent because the serovar has not been reported in human clinical leptospirosis or subclinical infection in Trinidad and Tobago but it has been documented in other countries [7, 36]. It is however pertinent to mention that all several panels of serovars used for MAT prior to the current study did not contain serovar Rachmati. 

 Regarding the seven serovars detected in the current study, five (Icterohaemorrhagiae Copenhageni, Icterohaemorrhagiae Icterohaemorrhagiae, Sejroe Sejroe, Ballum Ballum, and Bataviae Bataviae) have earlier been reported in human and animal infections or clinical leptospirosis in the country [26–28, 35, 38, 39].

## 5. Conclusions

 It was concluded that although the frequency of detection of serological evidence of exposure experience of *Leptospira* spp. in the students tested is relatively low, 20.3% by the ELISA and 7.1% by the MAT, in addition to the low titres (mostly 20 and 80) detected in MAT-positive samples, veterinary students have a significantly higher risk of becoming exposed to *Leptospira *spp. than nonveterinary students. None of the risks factors (age, year in programme, and gender amongst others) had any significant effect on infection by *Leptospira* spp. Serovar Icterohaemorrhagiae Copenhageni was the predominant serovar to which veterinary students were exposed while serovar Australis Rachmati was most frequent amongst nonveterinary students, with possible epidemiological implications.

## Figures and Tables

**Table 1:** Demographic data on students sampled for the study.

Factor	Number (%) of students sampled from FMS		
	School of veterinary medicine*	Other schools**	Total
Year in program			
1	28 (24.8)	19 (19.2)	47
2	32 (28.3)	34 (34.3)	66
3	28 (24.8)	22 (22.2)	50
4	14 (12.4)	16 (16.2)	30
5	11 (9.7)	6 (6.1)	17

Age (years)			
18	2 (1.8)	0 (0.0)	2
19	8 (7.1)	2 (2.0)	10
20	18 (15.9)	5 (5.1)	23
21	17 (15.0)	7 (7.1)	24
22	13 (11.5)	10 (10.1)	23
23	5 (4.4)	9 (9.1)	14
24	5 (4.4)	7 (7.1)	12
25 and over	21 (18.6)	58 (58.6)	79

Gender			
Male	29 (25.7)	28 (28.3)	57
Female	84 (74.3)	71 (71.7)	155

Residence			
Hostel	10 (8.8)	12 (12.1)	22
Apartment	42 (37.2)	30 (30.3)	72
Home	58 (51.3)	55 (55.6)	113
Home + apartment	2 (1.8)	1 (1.0)	3
Others	1 (0.9)	1 (1.0)	2

Diagnosis of leptospirosis			
Yes	0 (0.0)	0 (0.0)	0
No	113 (100)	99 (100.0)	212

Presence of pets			
Yes	91 (80.5)	57 (57.6)	148
No	22 (19.5)	42 (42.4)	64

Type of pets			
Home/pet	57 (50.4)	30 (30.3)	87
Guard only	7 (6.2)	8 (8.1)	15
Pet and guard	17 (15.0)	15 (15.2)	32

Contact with pet			
Low	23 (20.4)	55 (55.6)	78
Medium	31 (27.4)	25 (25.3)	56
High	53 (46.9)	11 (11.1)	64
Not applicable	6 (5.3)	7 (7.1)	13

Contact with livestock			
Low	6 (5.3)	1 (1.0)	7
Medium	16 (14.2)	1 (1.0)	17
High	13 (11.5)	0 (0.0)	13
Not applicable	78 (69.0)	0 (0.0)	78

Recent diagnosis in pets			
Yes	2 (1.8)	1 (1.0)	3
No	111 (98.2)	98 (99.0)	209

Rodent problem			
Yes	54 (47.8)	29 (29.3)	83
No	59 (52.2)	70 (70.7)	129

Farming			
Yes	12 (10.6)	2 (2.0)	14
No	101 (89.4)	97 (98.0)	198

Type of farming			
Livestock	11 (9.7)	2 (2.0)	13
Rice sugar	1 (0.9)	0 (0.0)	1

Pet rodent			
Yes	5 (4.4)	2 (2.0)	7
No	98 (86.7)	97 (98.0)	195

*Based on 113 students.

**A total of 99 participants comprising 46 dental, 39 nursing, and 14 pharmacy students.

**Table 2:** Frequency of detection of IgG immunoglobulins for leptospirosis by the ELISA.

School	Number of students tested	Number (%) with ELISA results	
		Positive*	Borderline**
Veterinary Medicine	113	11 (9.7)	19 (16.8)
Other schools***	99	1 (1.0)	12 (12.1)

Total	212	12 (5.7)	31 (14.6)

*ELISA concentration of 10 units and over.

**ELISA concentration of 5–9 units.

***A total of 99 participants comprising 46 dental, 39 nursing, and 14 pharmacy students.

**Table 3:** Titres of serovars of *Leptospira *detected by quantitative MAT.

Serovars	Veterinary students^a^					Other students^b^				
	Number of (%) positive at titres of					Number of (%) positive at titres of				
	20	40	80	160	320	20	40	80	160	320
Icterohaemorrhagiae Copenhageni	3 (2.7)	1 (0.9)	1 (0.9)	—	—	—	1 (1.0)	—	—	—
Sejroe saxkoebing	—	1 (0.9)	—	—	—	—	—	—	—	—
Sejroe Sejroe	1 (0.9)	—	—	—	—	—	—	—	—	—
Ballum Ballum*	—	1 (0.9)	—	—	—	—	—	—	—	—
Bataviae Bataviae*	—	—	—	—	1 (0.9)	—	—	—	—	—
Australis Rachmati	—	—	—	—	—	3 (3.0)	2 (2.0)	—	—	—
Icterohaemorrhagiae Icterohaemorrhagiae	—	—	—	—	—	—	1 (1.0)	—	—	—

Total	4 (3.5)	3 (2.7)	1 (0.9)	0 (0.0)	1 (0.9)	3 (3.0)	4 (4.0)			

*A sample had multiple agglutination.

^a^Based on a total of 113 students.

^b^Based on a total of 99 samples from 46 dental, 39 nursing, and 14 pharmacy students.

—: None.
